# The Role of Bedroom Privacy in Social Interaction among Elderly Residents in Nursing Homes: An Exploratory Case Study of Hong Kong

**DOI:** 10.3390/s20154101

**Published:** 2020-07-23

**Authors:** Aria C. H. Yang, Newman Lau, Jeffrey C. F. Ho

**Affiliations:** School of Design, The Hong Kong Polytechnic University, Hung Hom, Kowloon, Hong Kong; aria.h.yang@connect.polyu.hk (A.C.H.Y.); jeffrey.cf.ho@polyu.edu.hk (J.C.F.H.)

**Keywords:** privacy, social interaction, elderly residents, nursing home, indoor location tracking

## Abstract

Privacy is often overlooked in Hong Kong nursing homes with the majority of elderly residents living in shared bedrooms of three to five people. Only a few studies have used Bluetooth low energy indoor positioning systems to explore the relationship between privacy and social interaction among elderly residents. The study investigates the social behavioural patterns of elderly residents living in three-bed, four-bed, and five-bed rooms in a nursing home. Location data of 50 residents were used for the identification of mobility and social interaction patterns in relation to different degrees of privacy and tested for statistical significance. Privacy is found to have a weak negative correlation with mobility patterns and social behaviour, implying that the more privacy there is, the less mobility and more formal interaction is found. Residents who had more privacy did not spend more time in social space. Residents living in bedrooms that opened directly onto social space had higher social withdrawal tendencies, indicating the importance of transitional spaces between private and public areas. Friends’ rooms were used extensively by residents who had little privacy, however, the concept of friends’ rooms have rarely been discussed in nursing homes. There is evidence supporting the importance of privacy for social interaction. Future study directions include considering how other design factors, such as configuration and social space diversity, work with privacy to influence social interaction.

## 1. Introduction

The design of the physical environment has gained an increasing recognition as an important factor to facilitate meaningful interaction among older people with dementia living in nursing homes. Participating in meaningful social interaction with others is crucial in improving well-being and quality of life in people with dementia [1,2,3]. The traditional medical model for care, known to diminish social engagement, is no longer considered appropriate, and a new emphasis on person-centered or self-directed care is emerging [4,5]. Among these models, one central aspect of the change movement is the greater emphasis on autonomy, dignity, and, in particular, privacy. The advantages of private versus shared resident rooms has been a matter of debate in both hospital and nursing home settings, with some researchers and service providers advocating that the benefits of private rooms are self-evident, and others suggesting that private rooms are too expensive to build and operate [6,7]. The literature suggests that private rooms are of tremendous importance for older adults and that elderly residents overwhelmingly prefer private rooms over shared rooms in residential settings [7,8,9,10,11], even if it was smaller in size [12].

### 1.1. Privacy in Hong Kong Nursing Homes

Among the Hong Kong ageing population, a staggering 8% lives in institutional care facilities [13]. Hong Kong’s nursing homes mostly adopt the medical model for care which emphasizes on staff efficiency rather than resident interests and needs. These homes have strong institutional physical characteristics such as having a unit size of 30 or more residents, double-loaded corridors, shared bedrooms of 3 to 5 people, and large social spaces. By law, the minimum bedroom space for each resident is 6.5 sqm and many rooms in private homes fall below that standard to as little as 3 sqm [14]. With the increasing focus on implementing ageing in place policies, the overall residential care service will aim to provide care only for those with a higher level of nursing needs [15]. This implies that the nursing homes in Hong Kong will likely continue to adopt the medical model for care. 

There are 85% of community-dwelling older adults in Hong Kong that viewed nursing homes negatively, expressing feelings such as abandonment by their family members, insecurity, and loneliness towards nursing home placement [16]. According to a study [17], about 18% of the Chinese elderly residents living in institutional care environments suffered from depression because of a low level of socialization. Another study [18] discovered that Chinese elderly people in nursing homes were not open to socializing with the fellow residents—often avoiding or withdrawing from social interaction—out of the fear for conflict. The literature has well-documented frequent conflict between roommates in shared rooms over the use of television or radio, having curtains open or closed, having the door to the hallway open or closed, and air conditioning levels [7,19,20]. For Chinese elderly residents, the experience of living with others in an institutional setting was uncomfortable. They expressed the feeling of “walking on ice” when it comes to building relations with fellow residents [18]. 

When asked about their preferences towards privacy, Chinese elderly residents in Hong Kong expressed that privacy was unnecessary in residential living [18,21]. Chinese older adults living in nursing homes in Hong Kong were found to be deeply influenced by collectivistic traditions which encourage a greater emphasis on obeying rules and maintaining community harmony over individual privacy in nursing homes [21]. However, Chinese elderly residents also expressed strong preferences for private rooms and felt that having private rooms was highly associated with an improved quality of life [22]. A study of a group of residents who moved from traditional shared bedrooms to private rooms in Japan [23] found that even people who at first did not want a private room were highly content with their private room after the move. The reason for this discrepancy is perhaps due to the fact that opinions of elderly residents towards a shared room may have been expressed based on being somewhat accepting of their current situation in a shared room rather than being based on the experience in both a private and shared bedroom [7]. 

### 1.2. Measuring Social Interaction

Measuring the influence of a nursing home layout on social interaction is critical for the evaluation and reconsideration of traditional design principles of nursing homes. However, studies on this topic have one major shortcoming and that is the lack of continuous and unobtrusive means of measuring small group interaction in a nursing home. When it comes to evaluating the use of space in a physical environment, traditional methods such as ethnographic methods where researchers monitor the resident for an extended period of time, or photo diaries and surveys. Both of these methods are likely to render biased results, either due to the fact that participants may change their behaviour when they are being watched [24] or because they are inclined to provide socially acceptable responses to surveys [25]. In addition, studying the impact of a nursing home layout on social interaction can be complex due to the numerous variables that can impact behaviour. For instance, the resident personality and social network size may impact social interaction more significantly than the design of privacy.

This study was therefore designed to address these two issues. First, the study utilizes a dataset captured from a Hong Kong nursing home operator, collected via a smartwatch and Bluetooth Low Energy (BLE) system which is capable of capturing a large volume of information about a resident from a range of sensors [26]. The smartwatches are not intrusive and thus allowed the operator to capture the actual behaviour of the residents. Second, the study was conducted in a nursing home with different room typologies in Sham Shui Po area of Hong Kong. Data was collected in each of the shared room typology across five floors. Through a large scale deployment of location sensing technology in a Hong Kong nursing home, a set of location data of 50 elderly residents were provided by the service provider. The study analyzes the locations of residents from different room typologies and synthesizes spatial sequences to identify social interaction patterns the nursing home. 

### 1.3. Theoretical Framework

Our theoretical framework is guided by the work of Altman [27] on the influence of privacy on social interaction, but also with the analysis of location data collected by BLE systems. Building on previous studies [28,29], Altman defines privacy as a selective control of access to the self or to one’s group, it is an interplay among individuals and groups, implying that privacy means having control to open or close the self to others [27]. This line of thinking departs from the previous tradition which sees privacy as seclusion and avoidance of interaction with others [30,31]. Altman’s theory of environmental privacy mechanisms includes three methods, namely, density, objects, and territories, which are used by people to pace social interaction and achieve the desired privacy. 

Density refers to the number of individuals or groups who are involved in the social interaction and it is redefined by Altman as a privacy unit. In our study, privacy units are the number of elderly residents in shared rooms and individual floor space. The privacy unit is therefore the critical mass of dwelling density to avoid overcrowding and preventing the intrusion of personal space. *Objects* are the spatial interventions used by people to achieve the desired privacy. A study of family life in poor households shows that the parents would create their own private bedroom space with objects such as empty crates where they could be away from the children [32]. Privacy objects are also about defining personal spaces which are a series of distance zones appropriate for different social interactions [33]. Trespassing personal boundaries against the desire of a person may lead to potential conflict, tension, or discomfort, ultimately resulting in social withdrawal patterns [33,34]. 

Territories are interpersonal boundaries in the form of primary territory, secondary territory, and public territory. According to Altman [27], primary territories are the exclusive domain of an individual, secondary territories are represented by personal connections and not exclusive to an individual, and public territories are areas that are open to all. While the definitions of primary and public territories are clear, the nature of secondary territory has been the subject of further exploration. Secondary territories are now commonly accepted as transitional (semi-private and semi-public) spaces which provide a link between private and public spaces and naturally encourage social interaction in a neighbourhood setting [35,36]. Territories then are understood as a hierarchy of space with various privacy degrees: Private, semi-private, semi-public, and public space. In the context of nursing homes, the hierarchy of privacy is considered a key element in transforming institutional design into that of a household and that the inappropriate alignment of spaces—for instance, attaching a semi-public corridor to private bedrooms—would result in social withdrawal [37,38]. 

**Figure 1:**
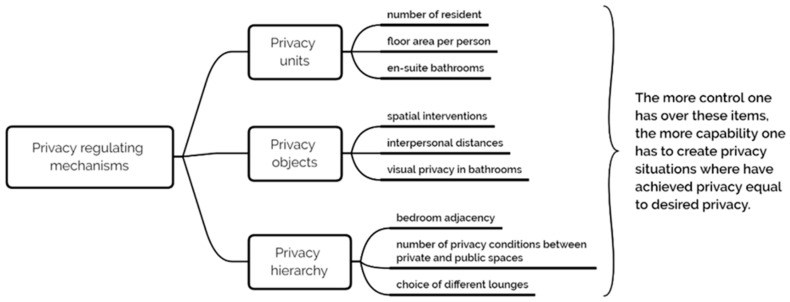
Framework for privacy design in nursing homes.

In summary, privacy is examined as three regulatory mechanisms (Figure 1) in our study: (1) Privacy units, the number of residents and individual floor space in a shared room and bathroom, (2) privacy objects, the number of spatial interventions used to define personal space among residents, (3) privacy hierarchy, the composition of spaces that follow the sequence of private, semi-private, semi-public, and public.

There are gaps in the current literature in regards to the relationship between privacy design and social interaction. First, there are only a handful of studies that have investigated the relationship between the design of privacy and social activities in nursing homes and most of them focus on the psychosocial outcomes such as preference and satisfaction. In view of this, the study aims to show the feasibility of analyzing the influence of privacy configuration on social interaction, adopting BLE systems, without the necessity of self-reported data from the participants. The second gap is that studies in sensor technology mostly focus on behavioural monitoring and pattern detection in regard to health status, lacking in discussions on social behavioural patterns in relation to privacy design. Very few spearheading studies have investigated the impact architectural configurations has on social behaviour using wireless sensing technologies [39,40]. This study also aims to evaluate the effects of physical privacy on social behaviour in a nursing home to support the extent of existing evidence that shows shared bedrooms diminish informal social interaction. 

There is a number of well-validated nursing home assessment scales, to name a few, Therapeutic Environment Screening Survey for Nursing Homes (TESS-NH) [41], Sheffield Care Environment Assessment Matrix (SCEAM) [42], and Environmental Audit Tool (EAT) [43]. However, they offer holistic standards for all shared rooms making it difficult to assess different typologies of shared rooms. This study also provides design indicators to evaluate privacy in individual rooms. 

### 1.4. Monitoring of Social Behaviour

There is an abundance of literature on behaviour-monitoring systems in the care of older people with dementia [44,45]. These systems commonly take the form of location-based sensing environments that capture an immense number of location information about older people from multiple sensors to detect behavioural patterns, behaviour change, and health status [26,46]. The technology has made ageing in place possible, enabling older adults to live at home more independently for longer while having their health status and real-time locations monitored by their loved ones and service providers [47,48]. The technology is also used to detect abnormalities in daily activity patterns of older adults and people with bipolar disorder using mobile devices [49,50]. 

In the field of architecture, a study used short-range lightweight RFID tags to detect face-to-face interaction among employees in an office building before and after renovation [39]. Another study by Ge’nois et al. [40] has used ultra-low power wearable sensors to detect face-to-face interactions for at least 20 s in an office building to track the social patterns of an epidemic spread. While there is a relatively higher accuracy in detecting social interaction using radio frequencies, this approach focuses on capturing only short-range exchange and limiting to detect social interaction in a large room. A different study [51] has used 17 BLE beacons at a range of 6 m to demonstrate how wireless location-tracking devices can be used to measure social interaction in the workplace. Through the collection of Received Signal Strength (RSS) logs, Montanari et al. [51] confirmed BLE’s suitability for detecting a prolonged proximity for the likelihood of an interaction at a 97% accuracy with 10 s granularity. However, there has been relatively less research utilizing BLE continuous location data to understand the social behaviour of elderly residents in the context of nursing homes. Furthermore, little study has extracted social interaction types based on the mobility patterns of elderly residents in nursing homes. Therefore, this study aims to further contribute to the application of BLE systems in the context of nursing homes through ubiquitous and continuous data. We also aim to utilize temporal and location data in a multi-user environment, for categorization and classification of probable social interaction patterns in accordance with spatial characteristics.

### 1.5. Impact of Physical Environment on Social Interaction

Research of nursing home design in environmental gerontology has extensively considered how the model for care and spatial configuration of private rooms in a nursing home could significantly improve residents’ social activities. There is also evidence that a private room is positively associated with an increase in psychosocial outcomes in nursing homes [7]. For example, it was reported that when residents with dementia moved from shared rooms to private rooms, sleep quality improved [52], conflicts among residents reduced, and the use of mental health medication was reduced [53]. 

More specific research in relation to bedroom privacy was found in environmental gerontology. For example, an extended study [54] compared social behaviour among residents who moved from a traditional nursing home with four-bed rooms on a double-loaded corridor to a prosthetically designed nursing home with private and double rooms. They measured behaviour change through questionnaires and behaviour mapping [29] and found that variations in bedroom privacy resulted in increased time in informal activity. The private room is associated with increased levels of informal activity [55]. Another study found that a high percentage of private rooms in a nursing home had a negative correlation with anxiety and aggression in residents with dementia [56]. In a study involving 15 residents, resident activity patterns were measured before and after a renovation of a long-term care unit and it was indicated that residents spent comparatively less time in their rooms and more time in motion and however, less social engagement. The study concluded that the reduced interaction may reflect greater choice over the interaction between private and shared rooms [10]. 

In architecture design principles, there are other useful methods for the analysis of such impact. For example, a study used space syntax to show that the design is a critical variable in overcoming a strong organizational structure, such as ones found in nursing homes, and facilitating unplanned movement and activity among elderly residents [57]. Another study in relation to building design in 30 German nursing homes similarly confirmed that the design of building layouts significantly influenced the movement of older people with advancing dementia [58].

### 1.6. Analysis of Social Interaction

Within a nursing home, there are varied understandings of how social interaction is represented among older people with dementia. In a study of informal social interaction, social interaction is defined as “a dynamic interplay between two or more individuals, where participants interpret and react to one another’s actions” [3]. Other studies [59,60] found that in the case of people with dementia, interaction may range from verbal expressions (e.g., short conversations) to non-verbal behaviour (e.g., eye contact, facial expressions, and coming into physical contact with others). The types of social interaction examined in this study include social participation (formal and informal interaction) and social avoidance (withdrawal and isolation). Informal interaction is particularly insightful because it is more likely for meaningful encounters to occur through more casual and day-to-day interaction among residents, as opposed to formal recreational activities [3].

These social behavioural types are related to privacy. According to Altman [27], when a person has control over his or her immediate environment (e.g., living in a private room), he or she will more likely choose to have social interaction with others. However, if a person’s privacy is intruded (e.g., living in a crowded shared room), he or she is likely to experience increasing tension and conflicts with the other involved parties and therefore will more likely result in social withdrawal. Another scenario happens when a person has too much privacy (e.g., living in a shared room at the end of the long corridor), he or she is out of contact with the others and thus is much more likely to experience social isolation.

**Figure 2:**
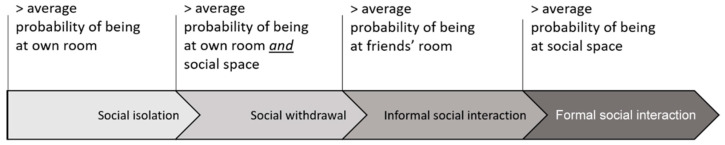
Definition of social behaviour.

For the purpose of this study, social interaction will be determined according to the resident location in “friends’ rooms”, “social spaces”, and “own rooms”. The proportion of these locations in a resident’s day will allow this investigation to calculate the probabilities of residents’ whereabouts and subsequently classify the type of social behaviour (Figure 2). Following this principle, a resident who spent a majority of time in friends’ rooms will be classified as someone who enjoys informal interaction; a resident who were largely located at social spaces will be classified as having the tendency for formal interaction; residents who spent a significant amount of time at social space *and* bedroom alone will be classified as having *social withdrawal* tendencies; and finally, residents who predominantly stayed in their own rooms will be categorized as having social isolation behaviour. 

In the following section, we investigate whether these differences in the four typologies of shared rooms are reflected in mobility and social behavioural patterns. We analyze resident mobility behaviour patterns based on the duration, frequency, distance, and timing of the location. We also identify social behavioural patterns from the spatial sequences. The research questions this study aims to answer are:

- What are the mobility patterns among the elderly participants and to what extent are the different levels of privacy reflected by these patterns?
- What are the social behavioural patterns among the elderly participants and to what extent are they associated with the different privacy levels in shared bedrooms?

In order to address these questions, this study analyzes a set of information collected from a BLE indoor positioning system in a nursing home in Hong Kong. The location data were visualized with various analysis of relationships, and then tested for statistical significance with respect to privacy design.

## 2. Materials and Methods

### 2.1. Location Data 

This study used a set of location data collected from a nursing home in Sham Shui Po area (Hong Kong). The nursing home had installed the BLE indoor positioning systems. With the collected data, an analysis of location data in relation to social behaviour were conducted. 

**Figure 3:**
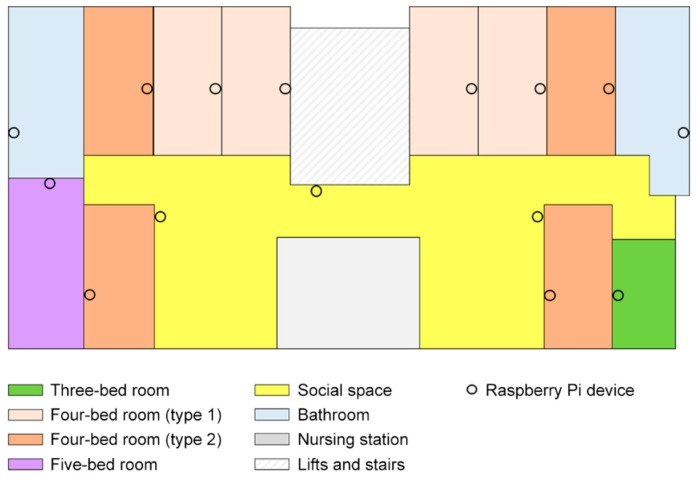
Typical unit floor plan with sensors. The figure shows a typical floor of a unit. Different colours represent the type of space in the home, e.g., social space, three-bed rooms, two types of four-bed rooms (type 1 faces the corridor, type 2 faces social space).

The configuration of the nursing home was designed in the form of the letter H; each unit had two subunits connected by a nursing station and pantry in the center (Figure 3). Each unit had one number of three-bed rooms, four numbers of four-bed rooms facing corridors (type 1), four numbers of four-bed rooms facing social space (type 2), and one number of five-bed rooms. For the experiment, 50 residents were recruited to wear a location wristband for 12 weeks. The systems were installed in five units on separate floors and ground floor reception area. Each floor was 416 sqm in size and contained 15 sensors (Figure 3).

The BLE devices have the same propagation characteristics as WiFi transceivers, both running in the 2.4 GHz license-free band. The BLE technologies provide indoor localization functionalities where each elderly resident wears a smartwatch that constantly checks for the presence of a Raspberry Pi (RPi). Each RPi, identifiable by a unique MAC address, is located in each bedroom, pantry, social space, and bathroom. The system determined each resident location by estimating the distance between a device and a RPi based on the Received Signal Strength Indicator (RSSI) of each signal. Upon receiving the RSSI, the server applies trilateration algorithms to locate the device by detecting the closest proximity from three or more BLE sensing points. The RPi were distributed with the goal where the majority of interior space is with a minimum signal overlapping to minimize environmental fluctuation. Through a series of testing and trials conducted in the lab and on site, we found that a high level of interference was caused by signal propagations (e.g., multipath fading, shadowing, fading) and environmental factors (e.g., building material and structure, fixture and furniture, and movement of people within the interior space). The results indicate that RPi worked best in a square room where possible. We also discovered that, depending on the location of each room and its surrounding construction material, each RPi signal strength, coupled with each smartwatch signal working as a RFID concept, needed to be finetuned individually to achieve minimal overlapping between devices, maximum position estimation, and minimal non-line-of-sight signal propagation. According to the smartwatch location estimates and space distribution records, an average accuracy of 85% was achieved in detecting resident locations. Given the limitation of the setup, the system gathered location information at any given time at alternating hours and of the whole 24 h period each day.

Of the 50 participants, six lived in three-bed rooms, seventeen lived in four-bed rooms (type 1), eighteen lived in four-bed rooms (type 2), and nine lived in five-bed rooms. The selected participants were relatively more independent with the capability to move around the nursing home and carry out daily activities of living. The study used a month of location data samples for analysis to ensure the variability of behaviour depending on the day of the week. In total, 18,200 location events were gathered. The 31-day location data of each resident were aggregated into hours, so each sample represented a complete day of 24 h. Of the total recorded location events, 15,336 (84.3%) were shorter than the 5-min interval.

### 2.2. Assessing Privacy and Topology in Shared Rooms

The typical floor is colour-coded to indicate the distribution of privacy in this nursing home. Privacy is studied in this investigation from the aspects of privacy units, privacy objects, and privacy hierarchy. An initial evaluation of the space shows that, of all the rooms, 80% are shared by four people, 10% shared by three people, and 10% shared by five people. The residents had an average individual floor area of 4.1 sqm. There are no en-suite bathing facilities and a public bathing facility is shared by 20 people in each subunit. In terms of hierarchy, the facility consists of semi-private bedrooms, semi-public corridors, semi-public bathrooms, and public social spaces (Figure 3). Considering these facets, various bedroom typologies can be identified. Typology 1 is three-bed rooms; typology 2 is four-bed rooms (type 1) which face onto the corridor; typology 3 is four-bed rooms (type 2) which face the public social space; and typology 4 is the five-bed rooms. The privacy conditions of these room typologies can be found in the detailed list of descriptions (Table 1).

As shown in Table 1, despite the architectural design, residents used their own efforts to maintain privacy. Rooms that were attached to the corridors and social spaces each had a set of half curtains at the bedroom door to block visual access from fellow residents and visitors. The only room that did not have curtains were the three-bed rooms that were located at the end of the corridor with semi-private pocket areas in front. In addition to the half curtains, all the doors were half closed. The beds were on average 1.1 m apart and there were no privacy curtains or partitions between the beds. The window curtains were drawn to block out sunlight, and personal clothing were placed on hangers by the beds and windows. These observations indicate that residents were protective of the privacy in their own rooms and had several tactics to block visual access into the rooms and beds.

However, in order to further quantify the privacy level in each room, these observations were not sufficient, and a set of assessment criteria was needed. Taking reference from some of the commonly used nursing home assessment scales such as TESS-NH, SCEAM, and EAT, a list of assessment criteria with geometrical indicators was compiled (Table 2). While all three are excellent scales, they could not be used to assess the privacy conditions in shared rooms because they considered all shared room typologies to be of the same quality despite configurations. To differentiate these shared room typologies, a nine-criteria list was developed based on the scoring principles of the assessment scales as well as relevant literature and were categorized according to privacy units (density), privacy objects (spatial intervention), and privacy hierarchy (spatial sequence) as proposed in the framework. 

### 2.3. Tracking Social Interaction and Behaviour

The study assumes that presence in a friend’s room is a good indication of meaningful social interaction, particularly, a location information within the distance of 2.5 m of a friend’s room (radius of the signal transmission range 5 m) for a duration of longer than 5 min. The data were aggregated into an hourly basis, and each location information is therefore a sum of the duration any resident has spent during that hour window. Therefore, this study is essentially capturing all aggregated interaction that have added up to be over 5 min at the same location in a given hour. In addition, the study focuses on social interaction in friends’ rooms, considering the shared rooms are small, only small groups of 2–3 people may gather which also sets small group interaction apart from larger group interaction in public areas. 

**Figure 4:**
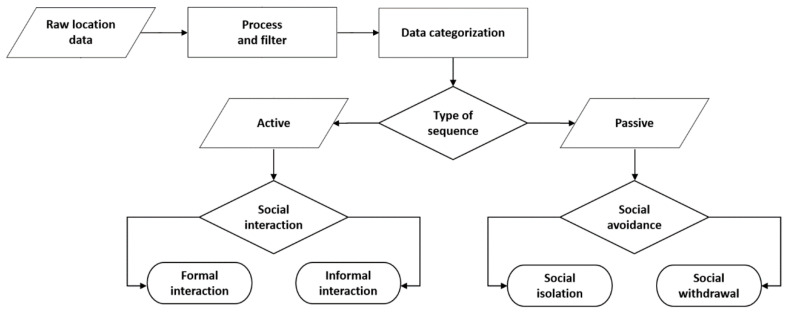
Schematic overview of social behaviour classification for the elderly residents.

The data were filtered and removed according to a process and a number of criteria to identify social behaviours (Figure 4). First, any location information that took place for less than 5 min were overlooked. Second, if a resident was found simultaneously in his or her bedroom as well as in the adjacent rooms or in the room directly above or below, that data entry is ignored. The filtered data were then categorized into location types of “own room”, “friends’ room”, and “social space” for the formation of individual sequential route by the hour. According to Montanari et al. [51], an average interaction is 1 min and 13 s long, with 70% of the captured interaction being shorter than 1 min while only 5% are longer than 5 min. Based on this discovery, the duration threshold is set at greater than 5 min to ensure that the captured interaction were more meaningful. 

Social behaviour patterns were then identified according to the sequence of locations. At any given hour, a resident could be engaged in two forms of social interaction: Informal or formal. For instance, if a resident’s sequential pattern is largely based in a friend’s room, then he or she is likely engaged in informal interaction while if a resident is mostly located in a social area, then he or she is likely to be involved in formal interaction. On the contrary, the resident could also be engaged in either social withdrawal or social isolation depending on the percentage of duration in the location sequence.

## 3. Results

The research resulted in one dataset, consisting of an aggregated duration of 50 residents in timestamped locations. No self-reported data through questionnaires nor interviews was conducted in this study. The goal was to test if the mentioned research questions could be answered without relying on self-reported data.

### 3.1. Mobility Patterns

Research question 1: What are the mobility patterns among the elderly participants and to what extent are the different levels of privacy reflected by these patterns? 

The study starts by examining the correlation between different privacy conditions and mobility patterns among residents. The analysis quantifies how much the privacy condition manifests in measured activities by calculating for a one-month tracking period the correlation between the privacy assessment scores and resident mobility patterns. A study found that mobility and social relationships are highly connected among elderly people [62]. In addition, Altman’s theory [27] of privacy and territoriality indicates that privacy influences mobility patterns which in turn impacts social interaction. Therefore, the study first examines the relationship between mobility patterns and social network size as well as privacy.

The Pearson correlation coefficient test is then conducted and expected to have two outcomes. (1) There is a negative correlation between privacy scores and mobility patterns outside of the bedrooms, suggesting that the more privacy there is, the less a resident would leave the bedroom unless it is for a meaningful interaction. Alternatively, if there is not a significant correlation between privacy levels and activity level, it would imply that privacy is not influential in determining the resident mobility patterns and meaningful social interaction. (2) There is a positive correlation between the number of social contacts and resident mobility patterns. In other words, the more friends the residents have, the more likely they will be engaged in social interaction outside of the bedrooms.

**Figure 5:**
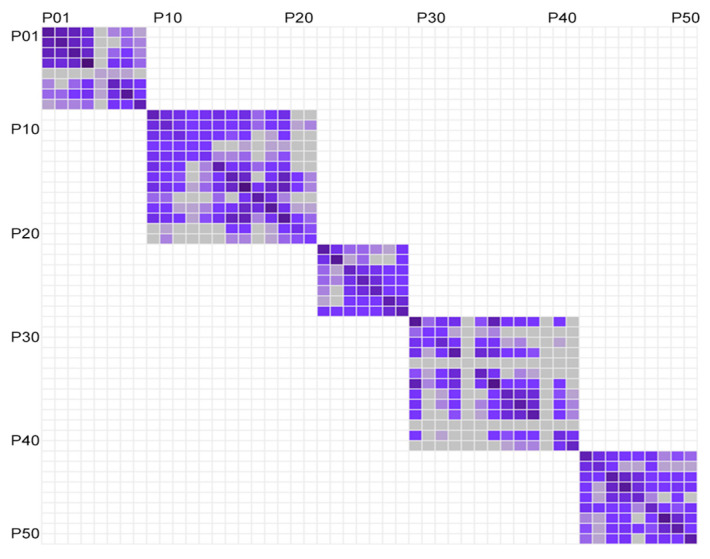
Netgraph showing inter-unit contact pairs in the five study units.

The study first investigates the social contacts of all 50 participants with one another in the nursing home. The analysis draws on incidences where paired residents were found at the same place at the same hour for over 5 min and identified them as social interaction. Participants belonged to five different units and they only had access to their own unit. Results reflect the circumstance showing that all the residents only interacted with residents of the same unit and no contacts were captured within other units (Figure 5). In the netgraph, each row and column represents an individual and the ordering of the individuals is arranged by units. The adjacent rows and columns signify residents of the same unit. A dark square indicates that the interaction was registered between the participants involved and a white square indicates no registered interaction.

The definition of mobility patterns refers to the resident movement within the nursing home in relation to the bedroom. The idea that bedrooms are of significant importance for residents’ social wellbeing reinforced that the design of nursing homes must start from the bedroom, rather than the entrance lounge area [37]. Thus, the study assumes that the bedroom plays the role as the origin of location and residents mobility patterns associated with the bedroom provides indication of the resident social lives and their relationships with the number of social connections and privacy. 

**Figure 6:**
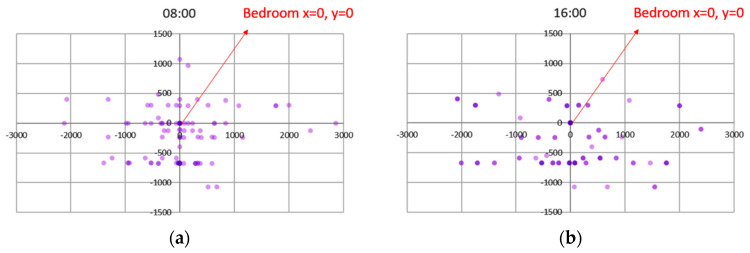
Resident and bedroom relationships. The collective movement patterns show that residents stayed around the bedrooms at 8 a.m. and moved farthest away from the bedroom around 4 p.m: (**a**) at 8 a.m., most residents were located within 5–10 m from their bedrooms; (**b**) at 4 p.m., some residents expanded their mobility range to between 10–20 m.

**Figure 7:**
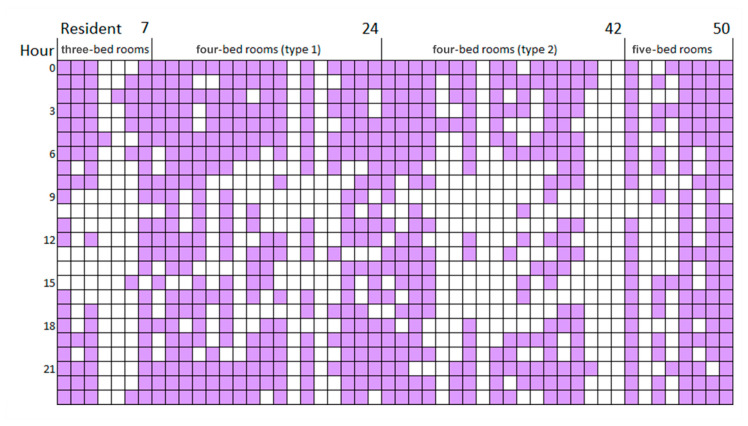
Individual movement patterns show that residents returned to their bedrooms regularly throughout the day. Purple squares mean that residents were located to be in their own bedrooms at a given hour while white squares represent residents were out of their rooms. For instance, residents of three-bed rooms did not go back to their bedrooms as often when compared with residents of other room types.

The scatterplot graphs (Figure 6) show that residents usually stayed close to their bedrooms in the mornings and gradually expand further out into the opposite sub-unit in the afternoon before returning to near-bedroom locations in the evening. This implies that distances travelled outside the room represents the residents’ intention to socialize, the farther away they are from the bedroom, the stronger the intention for social interaction. The analysis on the movement patterns shows that residents returned to their bedrooms regularly during the day (Figure 7). Dark squares represent the presence of residents in the bedroom and white squares represent absence of residents from the bedroom. Further analysis is conducted to analyze the distance travelled outside the bedroom based on the coordinates of each room in the nursing home in relation to the bedrooms and all the possible distances were calculated.

**Figure 8:**
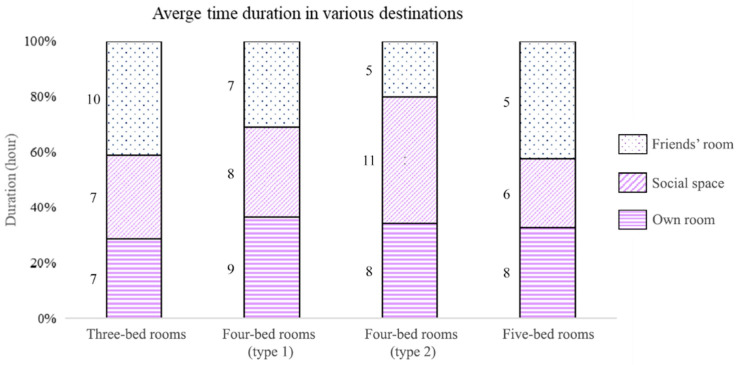
Average duration in various spaces. Both three- and five-bed room residents clearly spent more time in their bedrooms than residents of four-bed rooms. There are no indications that duration in friends’ rooms are affected by privacy levels.

The analysis of resident duration at a location shows that different privacy levels affect resident time spent in their own room as well as social space (Figure 8). Residents of three- and five-bed rooms stayed for an average of 10 h in their bedrooms, 3–5 h more than that of four-bed room residents and spent relatively less time in the social space. This indicates that more privacy is associated with increased duration in one’s own bedroom and reduced time in social space. However, residents in five-bed rooms also showed a tendency to stay in their rooms despite high density. It also appears that privacy does not have a clear effect on whether or not residents would visit their friends’ rooms.

**Figure 9:**
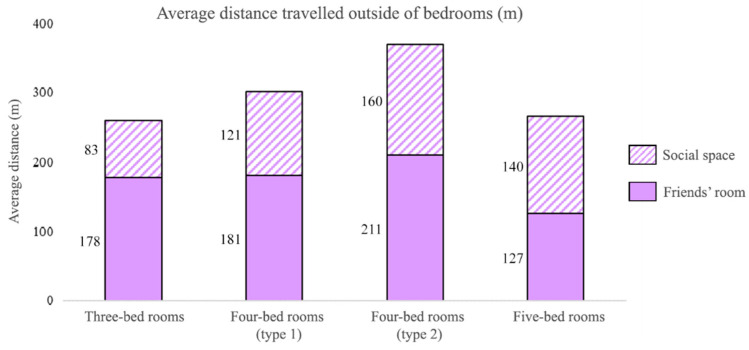
Average distance residents travelled outside of bedrooms. Residents in four-bed rooms (type 2) travelled the farthest to both social space and friends’ rooms.

The results of distance analysis (Figure 9) show that residents living in three- and five-bed rooms have noticeably lower travelled distances to social spaces and friends’ rooms, attaining only about half of those achieved in four-bed rooms. The highest distance travelled outside of the bedroom is found in residents of four-bed rooms (type 2) where there is not a great deal of privacy being next to the social space. The findings suggest that the privacy hierarchy has an impact on distances taken by the residents to the social area. 

**Figure 10:**
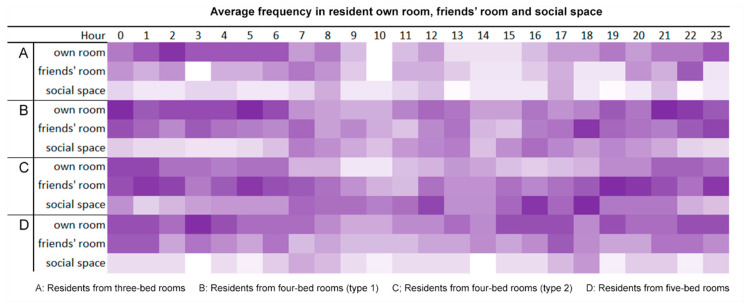
Average frequency of staying in resident’s own room, friends’ room, and social space throughout the day. Purple squares mean that residents were located in the corresponding room at a given hour while the darker purple squares represent a longer duration of staying there.

The number of visited locations per resident is illustrated through a colour-coded chart (Figure 10). The most active residents lived in four-bed rooms (type 2), with an average of 61 visited locations (highest 96 and lowest 2) over a 24-h time frame. Residents of four-bed rooms (type 1) were fairly active with an average of 55 visited locations with a rich range of colour sequences. Residents of five-bed rooms were relatively less active, having less varied sequential patterns located mostly in their rooms or friends’ rooms, with an average of 45 visited locations. Residents of three-bed rooms were the least active, with an average of only 36 locations.

**Figure 11:**
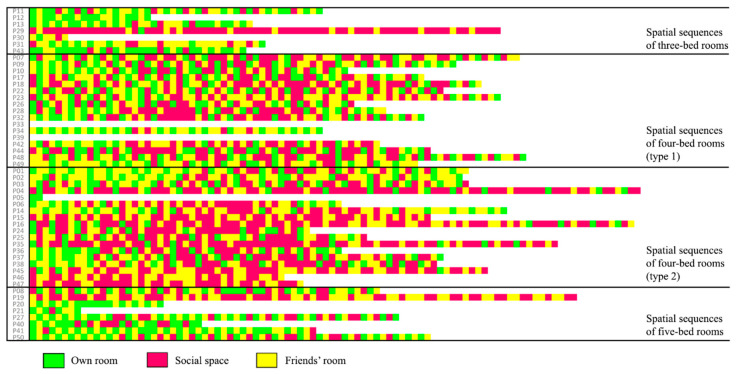
Colour-coded sequential patterns. This chart illustrates the number of visited locations per resident.

This study assumes that the level of activeness can be indicated by residents who were involved in more than 48 locations in one day (Figure 11), with the possibility of being more than two locations per hour. Table 3 shows that 14% of residents in three-bed rooms were actively engaged in different locations. While residents of four-bed rooms (type 2) had the highest average visited locations, four-bed rooms (type 2) had the highest percentage of active residents (82%). This analysis shows the privacy levels. 

Table 4 shows the value of Peasron’s correlation coefficient *r*, and the corresponding *p*-values, for mobility parameters of individuals in the nursing home versus the number of social connections and versus different privacy levels. Network size is positively correlated with residents’ movement to friends’ rooms with *r* = 0.36 (*p* = 0.01) for duration and *r* = 0.45 (*p* = 0.002) for frequency. Network size is also positively correlated with distances travelled to friends’ rooms by residents (*r* = 0.56). However, there is no significant correlation between network size and residents’ movement in relation to their bedrooms. Results suggest that there is a significant relationship between network size and informal social interaction, the more friends a resident has (larger network size), the more likely he or she will be engaged in informal social interaction. 

On the contrary, the correlation coefficient between different privacy levels and duration in friends’ room, *r* = −0.16, shows a weak negative correlation, but is not statistically significant, with *p* = 0.27. There is also a weak negative correlation between privacy levels and frequency of visiting friends’ rooms, *r* = −0.22 (*p* = 0.13). This investigation finds that privacy has almost no influence on mobility patterns.

### 3.2. Social Behavioural Patterns

Research question 2: What are the social behavioural patterns among the elderly participants and to what extent are they associated with the different privacy levels in shared bedrooms?

Social behavioural patterns are represented by resident locations in a series of spatial sequences composed of one’s own room, social space, and friends’ room. Further analysis was conducted to calculate each resident’s probability in various parts of the home (Table 4). As mobility results suggest (Figure 7), participants were mostly asleep between 9 p.m. to 8 a.m., this part of the analysis removes location data during that time to monitor only activity patterns in relation to the bedroom. 

Table 5 shows that three-bed room residents had the highest probability of being in the social space and least likely to be at the bed room, implying that they are more likely to be engaged in public social activities. Five-bed room residents had the highest probability of being at both friends’ rooms and bedrooms, signifying that they are more engaged in informal social interaction. This implies that the higher the privacy level, the higher participation in formal social interaction. On the other hand, the lower the privacy level, the higher participation in informal activities and in solitude. In terms of four-bed rooms, results show that type 1 residents had a relatively higher probability of being located at friends’ rooms than type 2 residents while type 2 residents had a higher probability of being at social space and bedroom. As an average, residents had a 35% chance of being at friends’ rooms, 43% chance of being at social spaces, and 22% chance of being at bedrooms. These findings suggest that residents were very likely to be engaged in either formal or informal social interaction during the day and that unit configuration appears to have an influence on the probability of a resident being at a given location. 

Of the 50 participants, 15 were categorized as having the tendency of formal social behaviour, 14 were reported of having informal social behavioural patterns, seven were identified as having social withdrawal tendencies, and 14 were classified as being in social isolation. Table 6 illustrates that the mean probability were used as multiple criteria to determine social behaviour and the percentage of each social behaviour cluster in each room type. Results show that five-bed room residents had the highest percentage of engaging in informal interaction and surprisingly the least percentage of social avoidance behaviour. Three-bed rooms had the highest proportion of residents participating in formal interaction, less participation in informal interaction, and relatively less residents in social avoidance. Findings also show that four-bed room types had relatively high proportions of residents who participate in social interaction, but they also hold the highest percentage of residents who are in social withdrawal and social isolation.

Pearson’s correlation was used to test the relationship between social beahviour type and privacy levels. Results show that the correlation coefficient (*r*) is −0.229 and *p*-value (*p*) is 0.003, showing that there is a weak negative correlation between privacy and social behaviour types.

## 4. Discussion

This study addresses the research gap focusing on the associations between privacy in the physical environment and social network size to influence social interaction among elderly residents in a Hong Kong nursing home. The study particularly adopted the use of BLE indoor positioning systems over the methods such as observations and self-reports to measure social interaction, directly comparing the mobility and social behavioural patterns of the residents from four shared room typologies.

This is normally difficult to conduct the current approach of study due to the sensitivity of using residents’ personal health and behavioural data for the experiment unless it was used in conjunction with the purposes of using ubiquitous technology solutions to revamp its model for care and improve the wellbeing of both the staff and residents. This study takes the advantages of the different bedroom layouts that are available on one single site which minimizes the challenge of accounting for all the possible variables that may impact the validity of the outcomes in comparison with studies that compare nursing homes in different kinds of buildings.

### 4.1. Theoretical Implications

The study provides evidence building on the body of work on this topic, supporting the concept of privacy could be important in facilitating social interaction among the elderly residents. The findings indicate that bedroom plays a significant role and is used by residents to control and maintain their privacy. The study confirms the work of [37] that bedrooms are at the heart of residents’ lives and more attention should be placed on ensuring the quality of bedrooms.

The study also sees that residents of three-bed rooms, having most privacy, were least active and spent much of their day within the bedroom. This contradicts with the findings in [10] stating that group residents spend comparatively less time in their bedrooms and more time moving about after moving to private rooms. The likely explanation for the differences in outcomes may be due to the fact that social interaction is not only determined by privacy but a combination of other design elements. Three-bed rooms being physically the farthest away from the social space and having no direct visibility and accessibility to other bedrooms and social area may discourage residents from leaving their rooms. This was reflected by the results that 86% of three-bed room residents stayed in mostly the same two locations every hour (Table 3). Researchers in [10] also noticed that these residents had reduced interaction with others, for which they argue that decreased interaction may reflect greater choice over interaction suggesting that residents were able to engage in more informal interaction in the privacy of their own room.

In terms of the two types of four-bed rooms; facing corridor (type 1) and facing social space (type 2), the findings align with previous studies. First, studies found that residents in hallway-based bedroom plans displayed less interests in their surroundings [63], as cited in [7], and a higher degree of lack in vitality and loss of identity [64]. This may explain that the residents in four-bed rooms (type 1) showed a preference of being in friends’ rooms rather than social spaces and a higher tendency of social withdrawal and isolation. Second, studies describe the benefits of having bedrooms opening onto the social spaces [52,63] and found that residents in this configuration had reduced anxiety and more interests in people and surroundings. On the other hand, while residents of four-bed rooms (type 2) demonstrated a higher level of social interaction, results also show that a greater number of residents in four-bed rooms (type 2) are in social withdrawal. This implies that an overly open configuration could render residents with little control over their privacy situations which may result in avoidance [27] and indicate that transitional spaces between the bedroom and social space could be vital for social behaviour. The findings also highlight that friends’ rooms are at a unique position to encourage informal social interaction to take place in-between the public and private areas. Results indicate that friends’ rooms are used extensively, especially by residents of five-bed rooms, and appear to act as a transitional space due to the absence of semi-private spaces on site. It can be interpreted that the high density in the five-bed rooms may result in more territorial conflicts and spatial intrusions [65,66] which may encourage behaviour in smaller group settings rather than large group interaction. However, the role of friends’ rooms has not been widely discussed in the literature and more studies could be directed into this area.

### 4.2. Limitations

The study has shown the use of sensor technology to track resident indoor locations and identify social interaction patterns in a nursing home. The dataset offers a possibility to provide insights for architects and nursing home operators to consider how the privacy conditions influence social behaviour. However, there are challenges associated with indoor location tracking. The technology’s capability to track an immense amount of location data in a nursing home may raise questions about individual privacy, how elderly residents would feel about being tracked at all hours, and whether this technology would encourage a change in behaviour. The precision of BLE systems is still to be achieved due to the fluctuation of WiFi signals. A recent study [67] points out that reflection, diffraction, and transmission loss around persons, objects, walls, and floors within the rooms can result in data loss or duplicated data on multiple sensors causing the accuracy of the location data to compromise. While it is likely that the experiment did not capture absolute numbers of locations, the aim of the study is to examine how the spaces of nursing homes are used for social interaction. It should also be noted that many more of such studies on nursing homes should be conducted to draw a general conclusion.

In particular, since this nursing home has a longstanding tradition of medical model for care (large unit and centralized nursing station), social interaction may also be hindered due to the care structure, implying that nursing homes that are of a person-centered approach or designs of a decentralized nursing station may be investigated in the future. The study also did not record the behaviour of care staff which is indicated as having a critical role in resident social interaction [3]. Furthermore, while the study has classified social interaction among residents according to whether it is likely to be of formal, informal, withdrawal or isolation, it was not possible for us to determine the content of the interaction due to the technology adapted. The emphasis here is to demonstrate that the lack of privacy could lead to social withdrawal and isolation and that the potential of implementing private rooms should be considered by service providers.

## 5. Conclusions

This study investigated a set of dataset of location traces captured using BLE indoor positioning systems for the identification of social interaction in a nursing home in Hong Kong. The analysis was conducted on how privacy levels and social network size of the care environment combined to influence social behaviour. Empirical evidence has reported the importance of privacy design for facilitating different types of social behavioural patterns. The results suggested that privacy is not simply a representation of density and spatial interventions but also a diverse non-linear hierarchical experience from public to private. Future studies could further consider how the other design factors, such as configuration and social space diversity, work with privacy to influence social interaction.

## Figures and Tables

**Table 1:** Privacy conditions in each room typology and measures taken to maintain privacy.

Room Typology	Floor Area (sqm) per Resident	Distance between Beds (m)	Bedroom Adjacent to	Objects Used by Residents to Achieve Privacy in Bedrooms
#1 Three-bed room	3.99	1	A pocket area along the corridor	No curtains were on the bedroom door. No objects were used between beds. Beds were not visible from the corridor or social area.
#2 Four-bed room (type 1)	4.38	1.2	Double-loaded corridor	Half curtains were on the bedroom door. Personal objects (towels, clothing) were hung between beds. Beds were visible from the corridor but not social area.
#3 Four-bed room (type 2)	4.38	1.2	Social space	Half curtains were on the bedroom door. Personal objects (towels, clothing) were hung between beds. Beds were visible from the social area as well as corridor.
#4 Five-bed room	3.79	1	Double-loaded corridor	Half curtains were on the bedroom door. No objects were used between beds. Beds were not visible from the corridor or social area.

**Table 2:** Design dimensions and indicators for privacy in shared bedrooms.

Dimensions	Definition	Geometrical Indicators
Privacy units	Assess how many people share a bedroom (TESS-NH; SCEAM).	3 people = 3
		4 people = 2
		5+ people = 1
	Assess how much floor area each resident has in shared bedrooms ([61]; SCEAM).	Above 12 sqm = 3
		7.6–12 sqm = 2
		under 7.6 sqm = 1
	Assess if there are en-suite washing and WC facilities in the bedroom (SCEAM).	Yes = 3
		Yes, but only washing area or only WC = 2
		None = 1
Privacy objects	Assess how privacy is achieved in shared rooms (TESS-NH; SCEAM).	Solid partitions = 3
		Privacy curtains = 2
		None = 1
	Assess the distance between beds in shared rooms [33].	120+ cm = 3
		45 < x < 120 cm = 2
		0 < x < 45 cm = 1
	Assess if there is visual privacy within bathrooms from helpers (SCEAM).	Alcove = 3
		Screen = 2
		None = 1
Privacy hierarchy	Assess which space is the bedroom adjacent to ([37,38]; EAT; SCEAM).	Semi-private space = 3
		Public social space = 2
		Semi-public corridor = 1
	Assess the number of transitional spaces (semi-private and semi-public) between the bedroom and social area ([37,38]; EAT).	2 transitional spaces = 3
		1 transitional space = 2
		No transitional space = 1
	Assess if residents have the choice of more than one lounge (SCEAM).	Yes = 3
		Yes, but it has more than four corners = 2
		No = 1

**Table 3:** Number of residents involved in more than 48 locations in a day.

Room Type	Three-Bed	Four-Bed (Type 1)	Four-Bed (Type 2)	Five-Bed
**Number of Residents**	1	14	14	4
**Percentage (%)**	14	82	78	50

**Table 4:** Pearson’s correlation coefficient *r* (and *p*-value) for mobility parameters versus social network size and versus different privacy levels. There is some positive correlation between mobility patterns and social relations. There is no significant correlation between privacy and mobility patterns.

Mobility Variables	*r* for Network Size	*r* for Privacy
Duration own rooms	0.17 (0.236)	−0.01 (0.960)
Duration social spaces	0.46 (0.001)	−0.13 (0.350)
Duration friends’ rooms	0.36 (0.010)	−0.16 (0.269)
Frequency own rooms	0.156 (0.279)	−0.054 (0.707)
Frequency social spaces	0.486 (0.000)	−0.155 (0.282)
Frequency friends’ rooms	0.425 (0.002)	−0.219 (0.126)
Number of social spaces	0.541 (<0.0001)	−0.262 (0.066)
Number of friends’ rooms	0.429 (0.002)	−0.109 (0.450)
Distances travelled to friends’ rooms	0.562 (<0.0001)	−0.184 (0.201)
Distances travelled to social spaces	0.364 (0.009)	0.015 (0.919)
Values in bold are different from 0 with a significance level alpha = 0.05		

**Table 5:** Probability of finding residents in the nursing home.

	Friends’ Room (%)	Social Space (%)	Own Room (%)
**Three-bed rooms**	33	49	18
**Four-bed rooms (type 1)**	39	38	23
**Four-bed rooms (type 2)**	32	43	25
**Five-bed rooms**	35	42	24
**Mean**	35	43	22

**Table 6:** Number of people engaged in a specific social behaviour in different rooms.

Room Type	Informal Interaction (%)	Formal Interaction (%)	Social Withdrawal (%)	Social Isolation (%)
	Friends’ Room > 35% and Social Space < 43% and Own Room < 22% OR Friends’ Room > 35% and Social Space < 43% and Own Room > 22%	Friends’ Room > 35% and Social Space > 43% and Own Room < 22% OR Friends’ Room < 35% and Social Space > 43% and Own Room < 22%	Friends’ Room < 35% and Social Space > 43% and Own Room > 22% OR Friends’ Room > 35% and Social Space > 43% and Own Room > 22%	Friends’ Room < 35% and Social Space < 43% and Own Room > 22% OR Friends’ Room < 35% and Social Space < 43% and Own Room < 22%
Three-bed	29	43	14	-
Four-bed (type 1)	35	24	18	24
Four-bed (type 2)	22	33	17	28
Five-bed	38	38	13	13
